# “It’s Like Youth are Talking Into a Microphone That is not Plugged in”: Engaging Youth in Disaster Risk Reduction Through Photovoice

**DOI:** 10.1177/10497323221136485

**Published:** 2022-11-09

**Authors:** Christina J. Pickering, Zobaida Al-Baldawi, Lauren McVean, Raissa A. Amany, Munira Adan, Lucy Baker, Zaynab Al-Baldawi, Tracey L. O’Sullivan

**Affiliations:** 1 EnRiCH Youth Research Team, EnRiCH Research Lab, 6363 University of Ottawa, Ottawa, ON, Canada; 2 Interdisciplinary School of Health Sciences, 6363 University of Ottawa, Ottawa, ON, Canada; 3 LIFE Research Institute, 6363 University of Ottawa, Ottawa, ON, Canada; 4 School of Epidemiology and Public Health, 6363 University of Ottawa, Ottawa, ON, Canada; 5 School of Community Services, 7964 Seneca College, Toronto, ON, Canada; 6 School of Psychology, 98607 Concordia University, Montreal, ON, Canada

**Keywords:** disaster, disaster risk reduction, climate change, youth, youth participation, youth engagement, photovoice, community-based participatory research, community resilience

## Abstract

Over the last decade, youth have been acknowledged as agents of change in the fight against climate change, and more recently in disaster risk reduction. However, there is a need for improved opportunities for youth to participate and have their voices heard in both contexts. Our Photovoice study explores youth perceptions of the capability of youth to participate in disaster risk reduction and climate change action. We conducted six focus groups from February 2019 to June 2019 with four teenaged youth participants in Ottawa, Canada, hosting two virtual Photovoice exhibitions in 2021. Our results highlight 11 themes across a variety of topics including youth as assets, youth-adult partnerships, political action on consumerism, social media, education, accessibility, and art as knowledge translation. We provide four calls to action, centering youth participation and leadership across all of them, to guide stakeholders in how to improve disaster risk reduction and climate change initiatives by meaningfully including youth as stakeholders.

## Background

Across the globe, humanity faces the growing challenge of extreme climate change and cascading disasters. Young leaders like Greta Thunberg, Autumn Peltier, Malala Yousafzai, Mari Copeny, and youth-led climate strikes are evidence that youth are passionate and vocal leaders in global efforts towards a more sustainable and equitable future. Beyond the individual level, there are youth-focused programs at the community, government, and international levels. For instance, a community-based program such as Preparing our Home (Preparing our Home, n.a.)—a project based in Canada to empower Indigenous youth as community leaders in emergency preparedness—designs projects with youth to build their capacity to lead disaster risk reduction (DRR). The majority of youth DRR programs in Canada are focused on education about preparedness and resilience, such as the school-based Master of Disaster Program from Emergency Management British Columbia (Cox et al., 2019).

In the Philippines, school-based science clubs are a space in which youth spread disaster knowledge between their school, family, and community (Fernandez & Shaw, 2015). There are also examples of non-DRR community programs which became integral to DRR in the face of disasters, such as the Vietnamese American Young Leaders Association who became an important resource for the Vietnamese community during the response and recovery from Hurricane Katrina in New Orleans (e.g., translating information for non-English speaking relatives, and raising community morale) (Mitchell et al., 2008).

Internationally, opportunities such as the United Nations (UN) Major Group for Children and Youth (UNMGCY) DRR Working Group—an international network of young people meaningfully engaged in UN processes—influence DRR at a policy level (United Nations Major Group for Children and Youth, n.a.). Despite some existing opportunities and evidence of youth leadership, children and youth lack structured, and sustained opportunities for meaningful participation in DRR and climate change decision-making and activities.

In 2015, three major global UN agreements were adopted: The Sustainable Development Goals (SDGs), The Paris Agreement on Climate Change, and the Sendai Framework for DRR. These UN agreements were designed to support action toward a more sustainable future, climate change adaptation, and reducing risks from disasters. The SDGs are a global agenda for action towards sustainable development (United Nations, 2015b); the Paris Agreement is an international treaty on climate change to combat climate change and invest in a sustainable low carbon future (United Nations, 2015a). The Sendai Framework for DRR is a global framework to reduce vulnerability to disasters; it emphasizes disaster preparedness and strategies to enable disaster recovery —and ultimately to foster resilience (UNDRR, 2015). There are synergies between these agendas, such as reducing risk across all hazards through a sustainable and equity-based approach to economic, social, and environmental development (Handmer et al., 2019). The global agendas focus on the need to enhance adaptive capacity, increase resilience, and limit vulnerability (Handmer et al., 2019).

Importantly, each of the three UN agendas emphasizes inclusive approaches and community engagement. The Sendai Framework stresses the need for inclusive measures to strengthen disaster resilience and an all-of-society approach to reducing disaster risk (UNDRR, 2015); the SDGs emphasize the need for participation across all stakeholders and people (United Nations, 2015b), and the Paris Agreement calls for the enhancement of public participation in climate change (United Nations, 2015a). The Sendai Framework takes these recommendations a step further by specifying how all-of-society partnership requires social empowerment through inclusive, accessible, and non-discriminatory engagement opportunities (UNDRR, 2015; Witvorapong et al., 2015). Similarly, Shaw (2012) states that sustainable community-based DRR strategies are dependent on partnership, participation, and ownership of local communities.

Children and youth are historically excluded from DRR and climate change decision-making and opportunities for engagement in policy and practice. At the same time, they are also considered a high-risk population in disasters due to their increased risk of experiencing negative health outcomes (Bartlett, 2008; Peek, 2008; UNDRR, 2015). Over the last decade, youth have been acknowledged as agents of change (UNDRR, 2015) in the fight against climate change, and more recently in reducing disaster risks (Amri et al., 2018), such as bridging the divide between youth and the UN system through international working groups (United Nations Major Group for Children and Youth, Disaster Risk Reduction Working Group [UNMGCY], 2016), or restoring mangrove ecosystems in the Philippines (Tanner, 2010). Aligning with Article 12 in the UN Convention on the Rights of the Child, which states that children have the right to participate in decisions that affect them and express their views freely (UN General Assembly, 1989), the Sendai Framework recommends the promotion of youth contributions and leadership opportunities in DRR (UNDRR, 2015). The difficulty lies in the implementation of these recommendations in practice at local, national, and international levels through programs that meaningfully engage youth in initiatives with real world implications.

Such engagement requires exploration of the capabilities and assets of individuals, communities, and organizations. Thus, the theoretical underpinnings of our research stem from the asset-based approach (Morgan & Ziglio, 2007), which highlights capabilities, over needs, when addressing inequities. The asset approach draws from the Theory of Salutogenesis (Antonovsky, 1996) which highlights how health is formed, rather than how disease is produced (i.e. pathogenesis). In the context of disasters, this approach focuses on assets that support resilience, and in the case of youth, how engagement can be facilitated by focusing on assets, resources, and capabilities.

Given the global guidelines advocating for inclusive, all-of-society engagement in DRR and climate change efforts, the purpose of this Photovoice project was to provide an opportunity for youth to participate in disaster research, while exploring their perceptions of youth capabilities in DRR and climate change action. Our article concludes with four calls to action to support stakeholders in engaging youth in DRR and climate change initiatives to create social change.

## Methods

### Study Design

We used Photovoice to engage and empower high school students and foster youth participation in action toward climate change and DRR. Photovoice is a community-based participatory research method intended to create space for community members to share their lived experiences and co-create knowledge with researchers (Wang, 1999; Wang & Burris, 1994, 1997). Using this method, community members are viewed as both participants and co-researchers with the understanding they are experts in their own lives (Latz, 2017). Co-researchers are invited to take pictures from their personal experiences and express their ideas through photo elicitation discussions on a given topic (Latz, 2017), and they are actively involved throughout every step of the Photovoice project. Given the collaborative, arts-based nature of expression, Photovoice is particularly useful for populations whose voices have been historically excluded from social change policy and practice (Wang & Burris, 1994), such as youth.

### Participants and Ethics

This study was approved for ethics by the University of Ottawa ethics review board. Consent was obtained from participants 16 years of age and over and parents of participants under 16 years of age, while assent was obtained from participants under age 16 years. All researchers and participants are members of the EnRiCH Youth Research Team (EnRiCH YRT), which is a community-based youth-led program where youth aged 13–30 years collaborate on DRR research and knowledge mobilization (Pickering et al., 2021). The request for a Photovoice study came directly from the youth team members of the community group. For more context about the EnRiCH YRT, see (Pickering et al., 2021). As a group of youth engaged in disaster preparedness and knowledge mobilization, they wanted their voices to be heard and to take part in a research study, where they have agency; this is one of the reasons we chose Photovoice as the method.

All study participants were recruited from the EnRiCH YRT. In total, we recruited four participants out of a possible 24 youth team members. The four participants elected to participate because they had the time in their high school schedules to accommodate the project, and they were interested in learning more about Photovoice, while having their voices heard on DRR. The small sample size is an appropriate size for focus group research as it allows each participant sufficient time to speak and permits in-depth data collection on the subject. Participants and researchers already knew each other, given their collaboration on projects in the EnRiCH YRT. This helped facilitate rapport and in-depth conversation during the focus groups. All four participants were active members of the community group, with several years’ experience in DRR. They participated throughout the entirety of our project, located in Ottawa, Canada, from February 2019 to June 2021.

In classic qualitative research terms, the four high school students would be called “participants”; however, given the collaborative nature of Photovoice intended to address the power imbalance between researchers and participants, the high school student participants are referred to as “youth co-researchers” for the remainder of the article. In our study, co-researchers are akin to actively engaged participants —as opposed to researchers trained in ethics or methods. This means, in addition to their role as participants taking photographs and participating in focus group discussions, they helped design the project, select photo assignments and topics for each focus group, provided member checking on codes and themes, wrote bullet points for the outline of our methods paper about the Photovoice project (Pickering et al., 2022), were consulted on all manuscripts providing feedback and approval on every draft, and actively led, designed, and implemented both Photovoice exhibitions. The youth co-researchers were 14–16 years of age when we started data collection; the University of Ottawa researchers included undergraduate and graduate students and a professor. Of the eight members of our team, four identify as non-white, three of whom are youth co-researchers.

### Data Collection

This study is part of a larger project where we hosted nine focus groups from 2019 to 2020 —six of them were in-person sessions in our research lab at the University of Ottawa from February to June 2019 (and are presented here). In 2020, we added three virtual sessions between June and August to discuss youth experiences of the COVID-19 pandemic—the data from those three virtual sessions will be published separately.

Each two-hour focus group session was attended by the eight members of our team. The principal investigators (CJP and TO) led each focus group asking the research questions and facilitating group discussions, while the two research assistants took notes. The four youth co-researchers responded to the research questions and shared their photos. We did not use the SHOWeD method typically used for facilitating focus group discussions in Photovoice (Wang, 1999) as the strategy was too formal and rigid to facilitate in-depth discussions for our group. Rather, we used more informal and naturally occurring probes such as “What else can you tell me about this image?” to maintain the natural flow of conversation. In this article, we present the results from the six in-person sessions, which focused on our views on youth engagement in DRR and climate change.

Our research protocol was similar to traditional Photovoice approaches from Wang and Burris (1994) (e.g., using focus groups to collect data), while incorporating modern techniques of community engagement, taking inspiration from Yi-Frazier et al. (2015) who modified the Photovoice methodology using Instagram to collaborate with adolescents. Before we began data collection, we identified research objectives, received ethics approval, and hosted a parent information session with potential participants. Our first focus group was dedicated to selecting the inaugural Photovoice assignment and explaining the rules about what could —and could not— be photographed (i.e., no pictures of other people or themselves where a person is readily identifiable). Given their advanced understanding of Instagram and photography, no photography training or exercises were necessary for our study group.

Between sessions, the youth co-researchers took pictures based on the assignment using their cell phones (with one participant opting to borrow a digital camera from the research lab) and posted their pictures to the private team Instagram account; the youth co-researchers were encouraged to comment on the pictures from their own cell phones, to facilitate discussion. Youth co-researchers also elected to use multiple art forms including photography, interactive art displays, lino block art, Word Art, and water color painting to express themselves. Meanwhile, the university researchers transcribed the audio-recordings from the focus group discussions and coded the data from previous sessions. The length between sessions ranged from two to 4 weeks, depending on everyone’s schedules, with the goal of completing data collection before the beginning of the upcoming school year. The decision to host six sessions was to enable the high school students to feasibly balance the project with their existing school and work schedules, while providing enough time to express themselves effectively.

**Figure 1.**
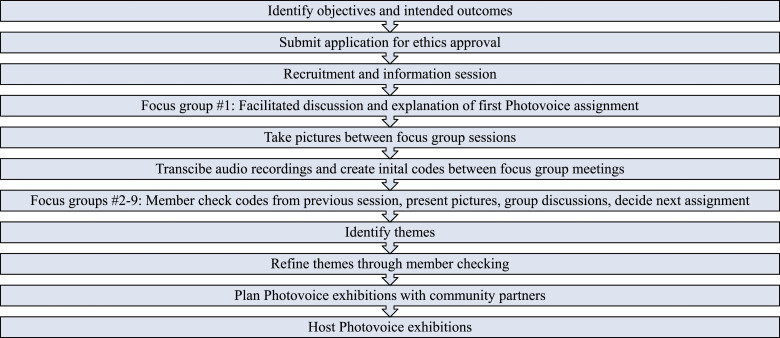
Our photovoice research protocol.

During the focus groups, the photographs were used to facilitate discussion. Each youth co-researcher took turns showing their photos to the group and explaining what the photo represented to them, and how they aligned with the photo assignment. The other youth co-researchers were then invited to discuss the photos and the meanings presented. This format often led to long, in-depth and organic discussions, rarely requiring probing questions from the facilitators. Our meeting agenda included the following: (1) checking codes with the youth co-researchers, (2) youth co-researchers sharing new pictures, (3) youth co-researcher group discussions about the pictures, and (4) selecting the next Photovoice assignment. We also allowed time for discussion related to planning the Photovoice exhibitions. Figure 1 summarizes our Photovoice research protocol.

### Data Analysis

We used reflexive thematic analysis (Braun & Clarke, 2019) to code data and create themes, using Word and PowerPoint to facilitate remote collaborative analysis throughout the pandemic. Reflective of the community-based, collaborative nature of Photovoice research methods (Wang & Burris, 1997), after the university researchers created codes and themes, we refined them through member checking during focus group sessions.

Throughout the analysis process, we protected confidentiality and anonymity by keeping transcripts and audio recordings separate, removing all identifying information from the transcripts (e.g., names), using password-protection on documents, and de-identifying quotations to enable youth co-authorship.

### Photovoice Exhibitions

Consistent with traditional Photovoice protocols (Wang & Burris, 1994), but applying online engagement strategies, we hosted two virtual Photovoice exhibitions with our community partners at the Canadian Red Cross to connect with community stakeholders. Community stakeholders included researchers and clinicians from Public Health Ontario, graduate students, professors, and university administrators, knowledge users from Ministère de la Sécurité publique and Public Safety Canada, and members of the Canadian Red Cross. These stakeholders are relevant to our study as they are knowledge users in DRR and public health, and experts in education, government or community practice. Our first exhibition was a public Instagram exhibition, available at the Instagram handle @yrtphotovoiceproject, which showcases the themes from all nine focus groups from 2019 to 2020. Our second exhibition was held as a workshop over Zoom at the *Disaster and Resilience Summit 2021*; our eight-member team, in collaboration with members of the Canadian Red Cross, presented a selection of themes to attendees and fostered interactive discussions on how to implement youth engagement strategies in policy and practice. The Canadian Red Cross was our community partner for both exhibitions.

## Results

**Figure 2.**
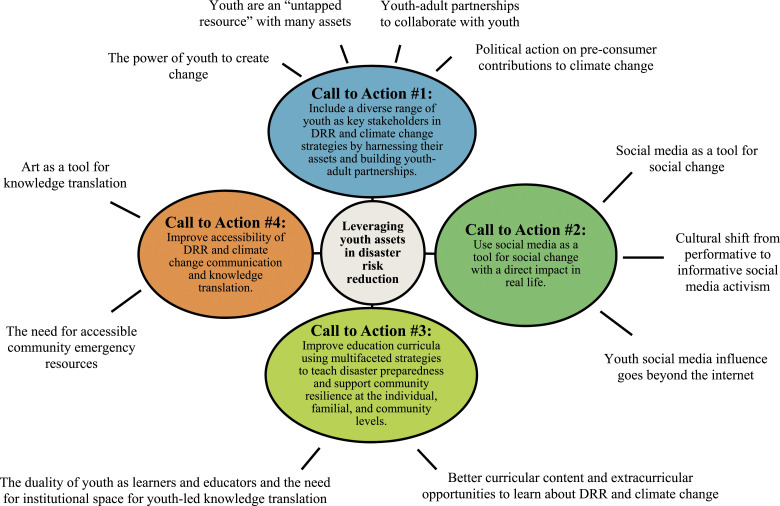
Overview of thematic results as they relate to four calls-to-action to create social change in DRR and climate change.

In this section, we present our results pertaining to the six focus groups held in 2019 and a selection of data related to discussions of social media from the 2020 sessions. The results are written from the perspectives of the youth co-researchers on our team—therefore the terms “we” and “our” in this section represent solely the voices of the high school student participants. Our results are divided into four calls-to-action for DRR and climate change, based on 11 themes covering broad topics, including the power of youth to create change, youth as assets, youth-adult partnerships, political action on consumerism, social media, disaster education, accessible communication, and art as a tool for knowledge translation. Figure 2 is a conceptual model summarizing the themes as they relate to our four calls to action for social change in DRR and climate change.

### Call to Action #1: Youth as Key Stakeholders and the Importance of Youth-Adult Partnerships

Our first call to action is to include a diverse range of youth as key stakeholders in DRR and climate change strategies by harnessing their assets and building youth-adult partnerships. We discussed four themes which led to this call to action: (1) The power of youth to create change; (2) youth are an “untapped resource” with many assets; (3) youth-adult partnerships to collaborate with youth; and (4) political action on pre-consumer contributions to climate change.

#### The Power of Youth to Create Change

**Image 1.**
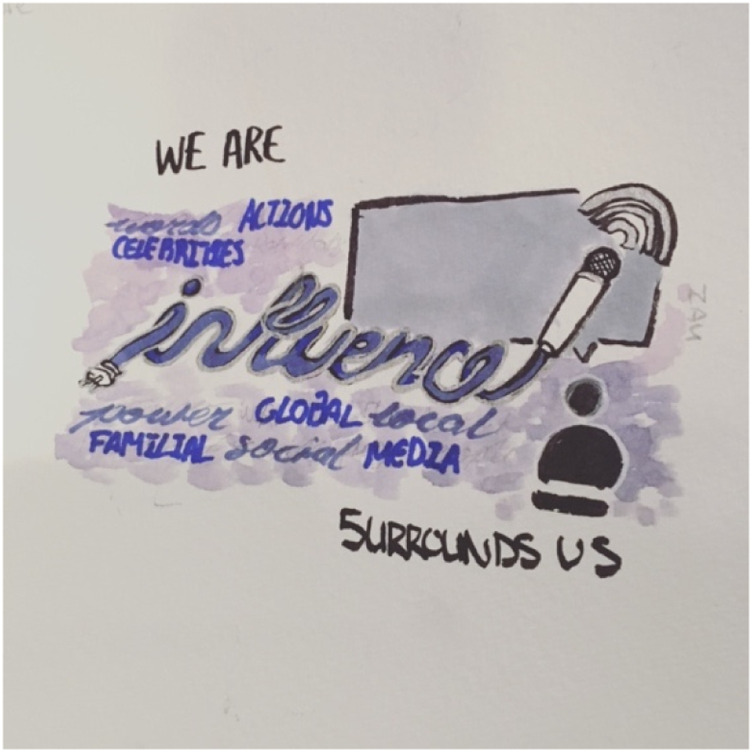
Word art created by a youth co-researcher representing the important influence youth have in their lives, which only accrues power through adult support.

Youth have a voice, but it feels “unplugged” (Image 1): “.*..it represents youth voices not being heard so even though youth are talking into the microphone, it is not plugged in so it is not going anywhere*.” (Session 4, April 27, 2019). Adults are not listening to youth as we voice our concerns and the need for immediate action on climate change; this is problematic because words precede action. We feel it is important for adults to reflect on how and what youth are messaging:

“*The way I am portraying influence here is… we can convince people through words... like people say actions are stronger than words, but things that prompt actions are usually words. So it is a double edged sword, and I [represented] that by having influence surrounded by words and influence being words*.” (Session 4, April 27, 2019).

Nothing will change unless we act. Everyone has some degree of influence which we can leverage to take collective action at all levels on climate change:

*“...I did a little word art with this … we are influence, influence surrounds us and I just put words around influence that I feel are synonymous with them... you have got global influence in terms of politics and local influence… and then familial influence… you and your family in the way you guys act and behave, the influence of your siblings’ action[s] and your own… And then social [influence] is just like you and your group of friends and you guys influence what you guys do and there is peer pressure and influence and media is influencing how people think…”* (Session 4, April 27, 2019).

Youth are essential assets in the fight against climate change and reducing disaster risk. Given appropriate opportunities, youth have the power and drive to reduce disaster risks and fight climate change. We are passionate about the power our generation inherently possesses, but we need adult support to meaningfully contribute.

*“... I recently went to this conference for the Ottawa-Carleton District School Board [organized, planned, and led by students from different schools] and so it was just a bunch of youth. All students came together and there was also a bunch of representatives and people in the school board that were high up and they were all listening to us. So youth came up on the stage… and they were telling their stories and you could see – they were really passionate* … *They were telling [the school board representatives] the change they wanted… I feel like we should go for it and go to our school representatives, people who are high up in power.”* (Session 2, March 2, 2019).

#### Youth are an “Untapped Resource” With Many Assets

**Image 2.**
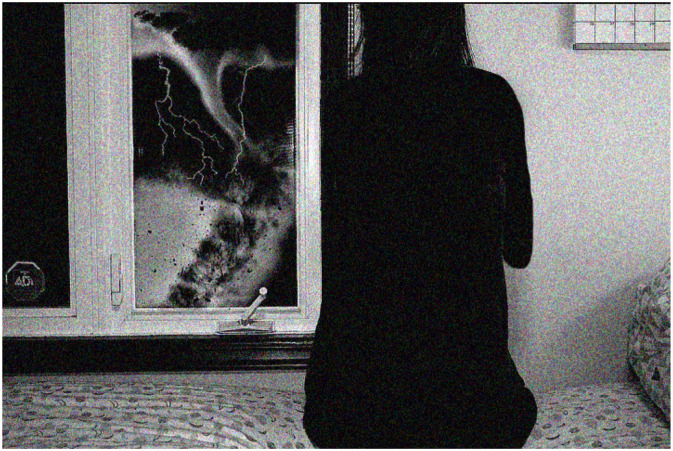
A young woman looks out the window, frightened by an impending disaster. The image represents the fear and urgency youth feel around the need to act on climate change and DRR.

As Image 2 represents, youth feel the urgency and need for climate change action and DRR, given the impact both will have on our futures. While today’s adults might not live to see the effects of climate change, youth are acutely aware that is the uncertain future we face: “*...this youth [is] looking out at disasters like ‘the disaster is there but only the youth can see it’...So this is youth looking out and seeing what is happening and adults are kind of like ‘oh what’s up?’*” (Session 6, June 1, 2019).

**Image 3.**
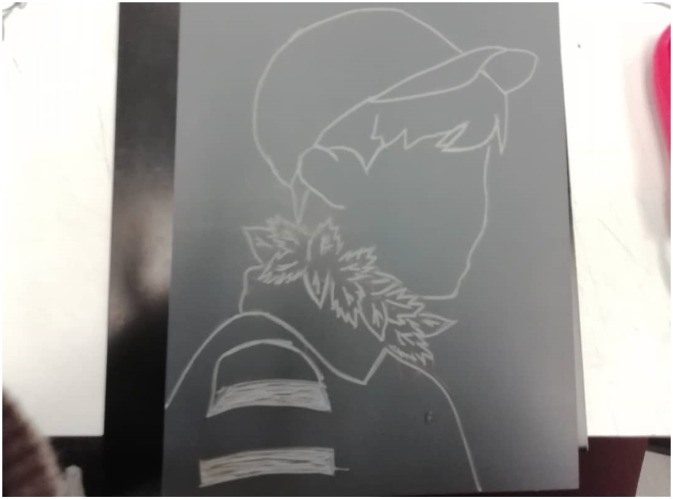
A piece of artwork created by a youth co-researcher. This carved out lino block represents the fear and anxiety youth feel around the effects of climate change and the need to engage in climate change action. However, youth are resilient —and we continue to collectively raise awareness and fight back despite these anxieties.

Despite this fear, youth are resilient and work together in the face of adversity. Image 3 shows a picture of a lino block, created by a youth co-researcher for art class. The purpose of the print is to symbolically depict climate change, something that we view as affecting our day-to-day lives. Despite the overwhelming emotions associated with the big tasks ahead, youth stand ready and resilient to face the problem head on:

*“... The print is of a boy (a youth) and around the boy’s neck there are leaves that are choking him. The symbolism behind it relates to climate change and the involvement and education about it. The boy represents the youth around the world and how rapidly the climate is changing and how that greatly affects our lives and how our future looks bleak. Although the leaves are choking him, he looks up and he is visibly resilient (not letting the leaves affect him). This represents how even though youth, and our future, is negatively affected by the rapidly changing climate and the consequences that stem from it (more natural disasters), we continue to raise awareness and work together in the face of adversity.”* (Session 4, April 27, 2019).

Given our strong feelings of anxiety and fear, and urgency to act, youth are strong assets in climate change and DRR initiatives. Youth action has the potential to contribute to DRR and climate change initiatives, if only adults took our contributions and capabilities seriously. For instance, the impacts from youth actions are already noted in the form of student-led clubs and school events:

“...*my school runs these events called coffee houses and they are student run. Students do everything and we raise money for certain charities. In this case, it did not relate to disasters, but it can… We made lots of money for the charity and it shows that we can [make an impact]*.” (Session 2, March 2, 2019).

In addition to our thirst for knowledge, and skills with new and emerging technologies, youth are creative, passionate, and bring diverse perspectives to the table. We are not a homogenous population—we mirror the diversity in the general community. Inclusion of diverse groups of youth in DRR and climate change awareness and education will reach high-risk, marginalized, and historically excluded populations in our community.

#### Youth-Adult Partnerships to Collaborate With Youth

While youth have the power to create change, meaningful contributions will be possible by partnering with stakeholders in DRR and climate change—leveraging each other’s assets and strengths to create innovations, opportunities, and change. Youth and adults have complementary assets; youth-adult partnerships provide opportunities to highlight potential contributions from everyone. For instance, youth are proficient with technology and social media; we can teach adults how to effectively navigate social media to improve disaster and climate change awareness. We bring different skills to the table and we are interested in being there; adults need to give us the opportunity for a place at the table, and take us seriously.

*“…even though youth are powerful and all, we do not have the same power as somebody that is a lot older and more experienced and also can really be politically involved without any restraint as to age or money so they are probably the most accessible, but the hardest audience to reach...”* (Session 4, April 27, 2019).

**Image 4.**
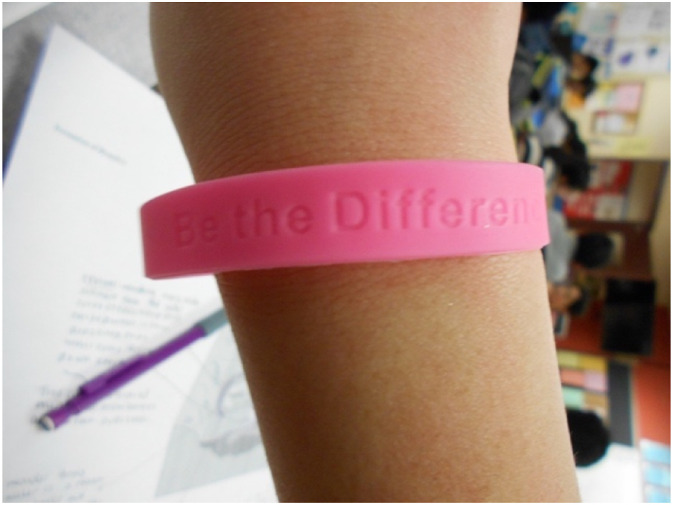
A youth co-researcher wearing a “Be the Difference” bracelet, representing the need for politicians to create youth-adult partnerships, to support DRR and climate change together.

We believe specific efforts are needed to build youth-adult partnerships with politicians so we can contribute effectively to DRR and climate change. Politicians and people in power have control over laws, policies, and practice. These are the stakeholders that youth want opportunities to connect and consult with. Image 4 represents the need for youth-adult partnerships in DRR and climate change action. Our message to politicians is: “*Listen and actually try and hear what we are saying – do not just brush us off*” (Session 3, March 30, 2019). Adults in power can listen and understand global problems from the perspectives of youth, while providing opportunities for youth to be politically active.

*“We always say that no one knows about us, we are an untapped resource and stuff, but a way to convey this is to go out to politicians and do activism and show how important disaster risk reduction and mitigation [is] and how it will influence us...”* (Session 3, March 30, 2019).

#### Political Action on Pre-Consumer Contributions to Climate Change

**Image 5.**
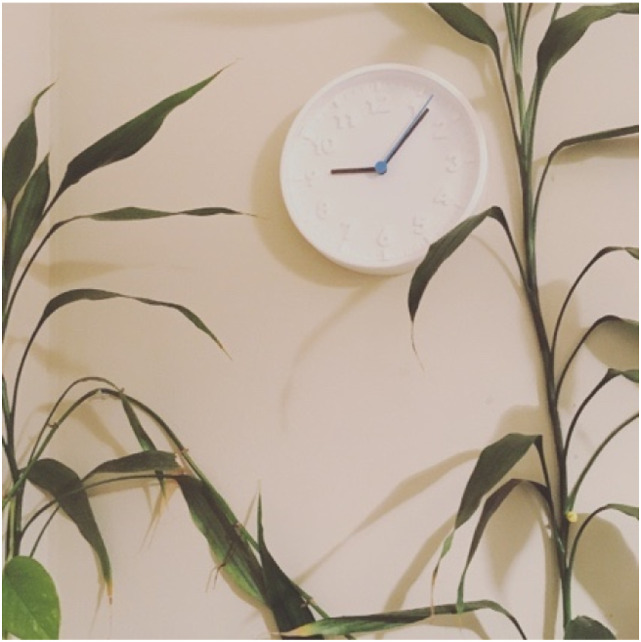
A picture of a clock and sparse plants, representing how humanity is running out of time to protect our dying environment from the effects of climate change.

Image 5 represents our feelings on climate change: *There is no Planet B*. We are running out of time to act on climate change. We feel the stress and responsibility of time moving forward and nature continuing to deteriorate.

*“We have until 2030 or 2050 to change the world so it will not come down crashing and burning because of what we did to it... this just represents like nature fading a little bit because of how sparse it is.”* (Session 2, March 2, 2019).

We discussed consumerism as an area in which politically based youth-adult partnerships can be beneficial in tackling climate change. On an individual level, thrifting is an example of a trend that reflects a cultural shift to responsible consumerism. Thrift stores previously were stigmatized — associated with poverty and lack of funds to buy clothes from fast fashion stores— but today, thrift store clothing is a fashion statement:

*“A couple of years ago, maybe like 2012 or 2013, if you go into a thrift store, it is because, it was for people that could not afford fast fashion, but like now… people go to thrift stores as a hobby... it is a big thing. There is a verb for it.”* (Session 6, June 1, 2019).

The verb for thrift store shopping is “thrifting”; it is seen as “cool” in its ability to save the shopper money, while owning style, and being eco-conscious:

*“If you go shopping at Value Village, you are helping the environment because new clothes do not have to be made. It helps you, because it is super cheap clothes, it helps Diabetes Canada because part of the proceeds go towards that. It is just a win-win-win situation, you know?”* (Session 6, June 1, 2019).

However, our biggest grievance with consumerism and climate change lies with fast-fashion companies. While changes to individual consumerism (e.g., shopping at thrift stores) are important, the ecological changes with the biggest impact will occur when big companies practice responsible consumerism. *“For a person throwing out one garbage can that day, a company throws out 70. So that is a really huge impact on the Earth considering that companies use a lot of the Earth’s resources.”* (Session 6, June 1, 2019).

**Image 6.**
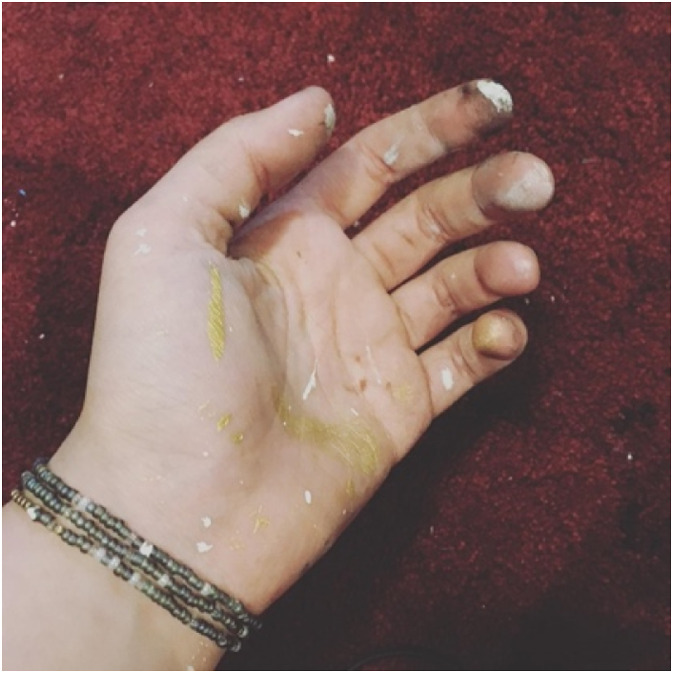
A hand covered in paint splatter represents how humanity already has the knowledge, resources, and tools at our fingertips to tackle climate change, we just need to apply them.

We want to see political action getting big companies to cooperate and make a difference on climate change. Companies have the power to change the fast-fashion cycle, reduce emissions, and landfill waste. Through youth-adult partnerships, we can tackle climate change using the resources already at our fingertips, as represented by Image 6. We have the tools we need to make drastic changes to the way humans live and companies produce; we just need to shift our priorities to apply our science and technology capabilities to climate change initiatives:

*“What I was trying to do with this hand is convey what we can do as humans, like our humanity to the world...we changed so much of the world in only a few hundred years, so if humans could do something for the better… humans could put that same energy from science and technology into giving a little bit more attention to the environment.”* (Session 6, June 1, 2019)

### Call to Action #2: Social Media as a Tool for Offline Social Change

Our second call to action is for stakeholders to use social media as a tool for social change with a direct impact in real life. Youth are an asset in harnessing the power of social media to effect change. Three themes emerged from our discussions and photos on social media: (1) social media as a tool for social change; (2) cultural shift from performative to informative social media activism; and (3) youth social media influence goes beyond the internet. The themes relating to this call to action differ slightly from the others as the data spans pre- and post-COVID-19 pandemic focus group sessions.

#### Social Media as a Tool for Social Change

**Image 7.**
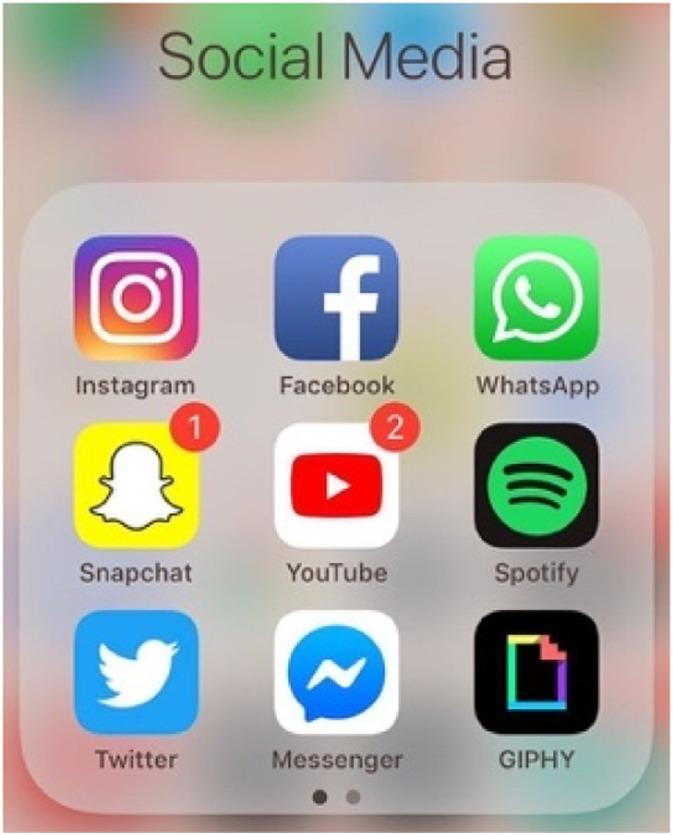
A screenshot of popular social media apps on a cellular phone, representing the reach of social media in our day-to-day lives.

Image 7 depicts a social media apps folder on a cellular phone, representing the prominence of social media in our lives. Social media can make disaster preparedness and awareness “cool”, creating a cultural shift in which it is “uncool” to be unprepared for disasters. This can be done through the influence of individuals with a social platform:

*“This is super unrealistic, but if Kim Kardashian was like ‘oh just made my disaster preparedness kit using like Supreme’s disaster bag’ then everyone would be like ‘oh my god I need this.’ The whole culture of influencers can totally change the mainstream, what is cool, literally overnight...”* (Session 3, March 30, 2019).

Social media is a strong resource to raise awareness of DRR and climate change because it is a fast channel for communication that youth are skilled at using:

*“A lot of people say this and it is really true how social media is one of the biggest resources to spread awareness... For example, stuff spreads really fast, like the Kim K. drama that is going on right now... something could happen and it is instant. I just want to say we should really be [using it], for disaster risk stuff and also for climate change, it is a really good resource, and also youth know a lot about it.”* (Session 2, March 2, 2019).

Social media is also a strong communication platform given message framing and distribution of power. Social media platforms, such as TikTok, are an excellent source of information sharing for youth because the model presents information in a digestible manner and uses humor to reach and relate to the audience; this framing is a contributing factor to its popularity.

Additionally, social media applications like TikTok use an algorithm that more evenly distributes power in the hands of all users, as opposed to a select few. This allows for increased information sharing from non-celebrity youth to reach wide ranging audiences and effect change.

*“Plus, in what you mentioned about TikTok… it is an extremely good source for sharing information because, unlike YouTube, [which] just pushes things out based on how popular the content creator was before, [TikTok] pushes out anything possible. It is a genuinely good place to find content, especially content for activism. Plus, TikTok has a way of wrapping its message up in comedy so it makes it a little bit more like easily digestible.”* (Session 7, June 17, 2020).

*“And also something I have noticed is that a lot of the popular creators are made popular by actual people on the app and like youth. So I feel like that is another contributing factor”* (Session 7, June 17, 2020).

The more even distribution of power influences our trust in social media over news media for reliable information. People are becoming more conscious about where they get their sources of important information such as reports on BLM protests, or COVID-19. We prefer social media over news media to access information because it feels more truthful, given the more equitable distribution of power, compared to news media. Information on social media feels less biased than news media.

*“... I have seen a lot of opinions going around saying like, ‘oh, I can only trust social media right now’ because the news they are not really showing what is happening, which is kind of true because, there are threads on Twitter about police inflicting violence on [BLM] protesters. But if you look at the [news] media they are talking about how rioters are the ones that are creating violence and burning down buildings. But if you look at social media, it is [produced] by the people – it is not really easy to skew it*…*”* (Session 7, June 17, 2020).

However, social media should be used with caution. Just because someone “shares” a social media campaign, does not mean any real action occurs offline. Some social media users will interact genuinely with online campaigns, while others interact superficially to avoid being judged negatively by their peers; it is difficult to distinguish between the two. For social media to work as an awareness tool, there needs to be a direct impact in reality:

*“...I wanted to critique a little bit how social media, although it is a really good resource to spread awareness… We should not think that we have done enough just because we have shared something...”* (Session 2, March 2, 2019).

#### Cultural Shift From Performative to Informative Social Media Activism

Social media shaped our upbringing—we are the digital generation: *“...we grew up, all of us, with media. Especially millennials and Gen Z’s so I feel like, in a way, media has already shaped us from the beginning…”* (Session 4, April 27, 2019). With this upbringing, what we saw happening with social justice campaigns on social media in 2019 was performative, static information, and lack of action. At the time, social media was being used to spread awareness, but there was no real action. Some campaigns existed only to follow the pressures of social media trends, without necessarily following up on the actions they pretend to promise for the publicity—they are simply performative:

“*As you can see there are static pictures at the front so it is… like people posting on Instagram like ‘oh if you like this post you will plant trees’ so you are trying to spread awareness, but there is no real action to it. More like static information that you pass on to other people because people keep posting them and then there is no real action taken. We do not know that they are actually going to plant trees [from this campaign]*” (Session 4, April 27, 2019).

**Image 8.**
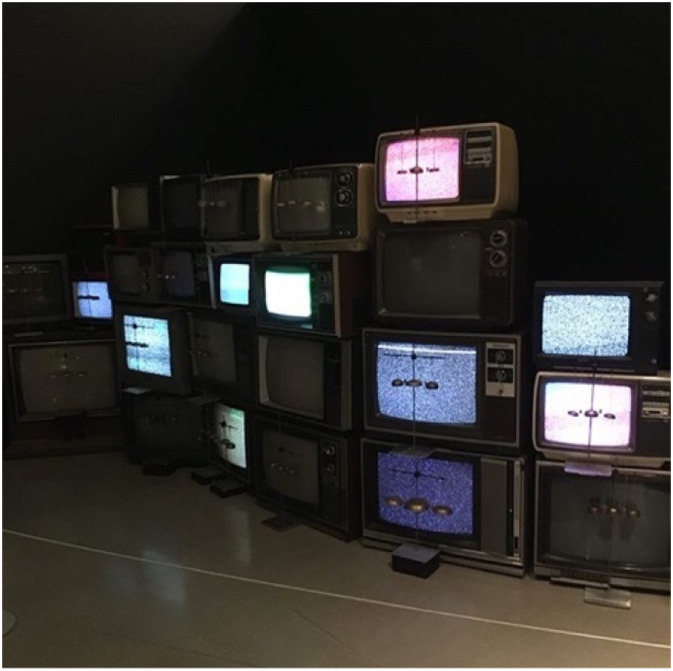
Old tv boxes with static images in an art exhibit atthe Ottawa Art Gallery, representing performative activism through static information sharing on social media platforms. (Art installation credit: Darsha Hewitt, Electrostatic Bell Choir, 2013, Electromechanical sound installation, cathode ray tube televisions, bells, pith balls, metal, electronic control system, Carbon + Light: Juan Geuer’s Luminous Precision at the Ottawa Art Gallery).

Our overall perception about how people use social media changed in light of the context of COVID-19, BLM and the crisis in Yemen. Social media posts began trending away from performative activism to informative activism. A performative social media presence can be situated within performative allyship—it involves users passively sharing information with the intent to protect their social image, rather than to contribute meaningful action to a cause (Image 8).

An informative social media presence is genuine online engagement with a cause, as indicated by what a user is sharing and long-term sustainment with the cause. For instance, in 2020, social media users leveraged online platforms to move beyond statements standing in solidarity with a cause:

*“...they are sharing more petitions and more impactful things other than, say [just] the ‘standing in solidarity’ [post] – even though [those posts are] important too. I feel like social media has become a more important tool [during the pandemic].”* (Session 7, June 17, 2020).

Thus, it is now considered “cool” to be overtly socially conscious by using your social media presence to call out non-compliance of pandemic guidelines (e.g., not wearing masks) and share impactful information (e.g., petitions against police brutality). We identified several reasons for this cultural shift. First, people were angry and scared because of the uncertainty around the pandemic, and second, the compounding effects of cascading disasters and global social movements made citizens emotionally responsive. Third, citizens relied on social media to stay connected to rapidly changing news, and to their social networks during the pandemic. The outcome is that emotionally charged citizens were regularly exposed to emotionally charged social issues, resulting in more active online community engagement and pressure to take action.

*“People are actually starting to get more angry and they are starting to see it more as being a flex to be woke. People are genuinely starting to step up and be like ‘yo, something is wrong’ and trying to educate people. People are not taking any crap anymore, sort of because it is the BLM [movement] and the [COVID-19 pandemic] coming so soon together. People are just starting to get more pissed that nothing is happening. People are starting to notice... this is an important issue... because it is really taxing to be around that environment for a really long time.”* (Session 7, June 17, 2020).

*“But what I have noticed the past couple of weeks is that people share a lot on their… Instagram stories, or through TikTok, video after video about what is happening right now. … what I think it helps to do is to create pressure. If you are bombarded with all these posts, and all these opinions and statistics all the time, you are pressured to look at it and you are pressured to care … So you cannot, especially [with] COVID-19, you cannot really turn off your phone and be oblivious because you use social media now to communicate because you cannot really go outside and talk to people. And you cannot go to school. So seeing that all the time, like bombarding your face or pressure to look into it, and read about it, and take action.”* (Session 7, June 17, 2020).

#### Youth Social Media Influence Goes Beyond the Internet

Being “woke” (aware of social justice issues, especially racism) can take place on social media and in day-to-day practices and conversations offline. Youth are taking conversations beyond social media and trying to incite changes in their lives. They are taking actionable steps by addressing social justice issues —by educating those around them about the information they learned online. In this way, youth are acting as knowledge brokers, transmitting and explaining what they see on social media to family members who do not have an online presence.

*“… sometimes it is not only what you see on social media that is creating an impact but what people take away from [it] and what they say to their family members or what they educate them [on]. There is more impact being done beyond social media.”* (Session 7, June 17, 2020).

*“Like when my parents [and grandma] asked why they were protesting, I explained to [them] how George Floyd was killed and what was going on. At first, my grandma took the side that the rioters are bad because of the violence, which by the way was started by police officers. So I told her and she started to be more empathetic about the protestors. So I felt like that was a good change.”* (Session 7, June 17, 2020).

### Call to Action #3: Disaster Preparedness Education to Support Community Resilience

Our third call to action is to improve education curricula using multifaceted strategies to teach disaster preparedness and support community resilience at individual, familial, and community levels. Education is an important area in which youth can affect change on DRR and climate change. Two themes emerged from our discussions on how to leverage the education system in DRR and climate change: (1) Better curricular content and extracurricular opportunities to learn about DRR and climate change; and (2) the duality of youth as learners and educators and the need for institutional space for youth-led knowledge translation.

#### Better Curricular Content and Extracurricular Opportunities to Learn About DRR and Climate Change

**Image 9.**
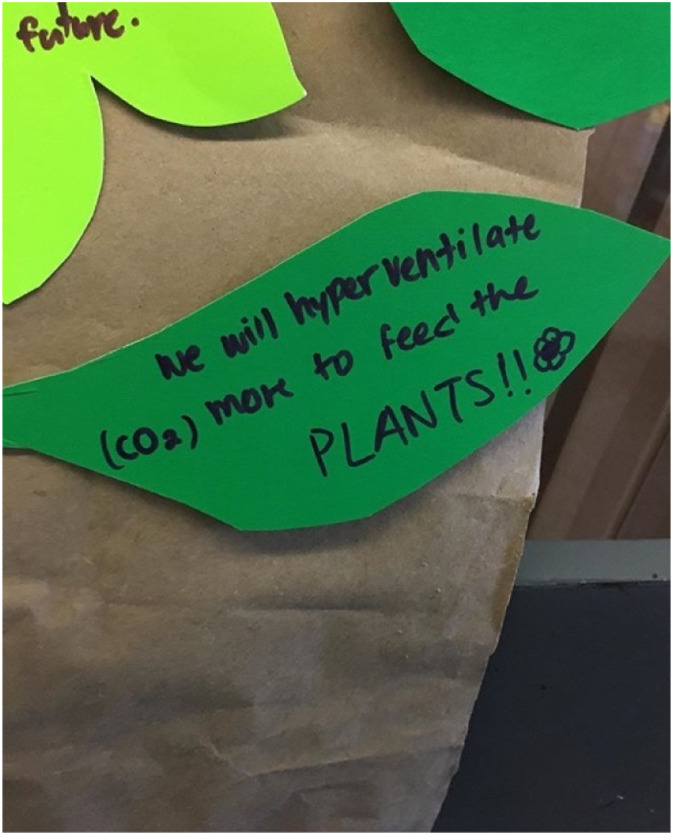
An interactive art installation in which students added their own thoughts on climate change.

Existing content in educational curricula is insufficient to teach practical preparedness skills and DRR knowledge. Image 9 shows an interactive art installation at one of the youth co-researcher’s schools. They were asked about their thoughts on climate change—an example of a fun school-based initiative to discuss climate change. DRR content needs improvement and expansion for students to gain these skills through a combination of interactive and exciting strategies.

*“... Because we know what to do during fires and we know what to do during other stuff like that, but we do not know what to do during an earthquake or like a hurricane. So if schools were to dedicate a week or maybe a few days of a certain month [to disaster education]... I was a little bit disappointed with the lack of how they talked about preparedness for disasters because we only did a project about disasters, but not what to do after a disaster.”* (Session 5, May 11, 2019).

*“...and make it fun. Because often times when [it is fun], students tend to talk about it. Like a fun trip or a fun little event, or something unique, students remember it and they talk about it and it stays in their mind. Because day to day classes, you take that information and you use it on the test, and then you dump it out.”* (Session 5, May 11, 2019).

Through improved curricular content and greater extracurricular DRR and climate change opportunities, the education system can reach hard-to-reach populations on these topics. There is a need to address the knowledge gap most individuals have regarding disaster preparedness, especially for youth who might be hard-to-reach. The education system infrastructure is an asset that can be mobilized to reach this population: “*...we can have a bit of an emphasis on disaster relief [in schools to reach students living in low income neighbourhoods]*.” (Session 5, May 11, 2019).

#### The Duality of Youth as Learners and Educators and the Need for Institutional Space for Youth-Led Knowledge Translation

**Image 10.**
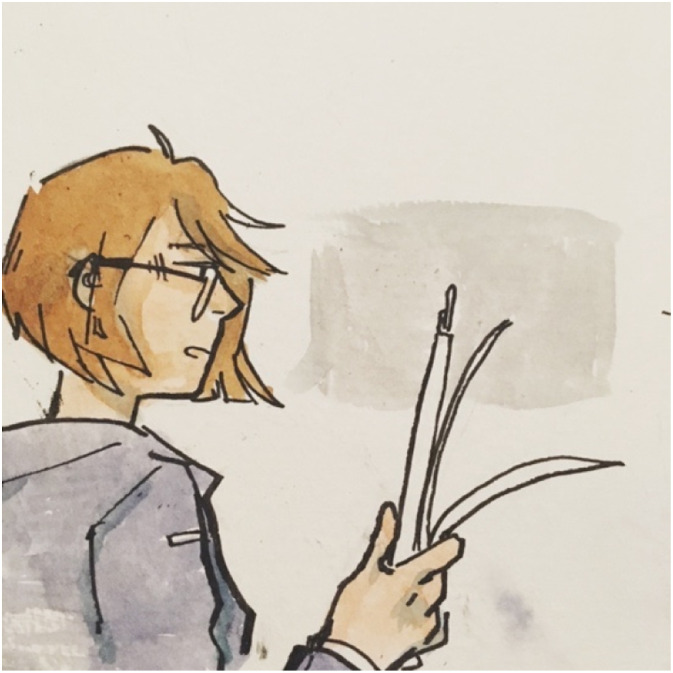
Artwork by a youth co-researcher depicting a young woman passing her DRR and climate change knowledge on to others, representing youth as community educators.

Youth can and do exist in dual roles as learners and educators. The artwork in Image 10 depicts a young woman sharing her DRR and climate change knowledge. Proper DRR and climate change education in schools can provide youth with a strong foundation to stand on our own as community leaders, thus raising awareness in our communities. The next step is to provide youth with platforms to apply and disseminate this knowledge: *“This is a youth researcher … I decided to go through the knowledge route. [She is holding] papers… doing research and presenting the information, hence the speech bubble in the background. [She is] passing her knowledge on”* (Session 4, April 27, 2019).

One way to support youth as educators in the community is to provide the institutional space for youth-led knowledge translation opportunities, such as youth-led conferences. Youth-led conferences could involve peer mentorship and learning amongst youth, supported by professionals. Learning could occur through hands-on workshops that allow youth to actively interact with disaster risk science and climate change. When youth have the opportunity to attend and lead conferences, they gain new skills which they can then share with their friends, family, and community: ***“*** *You do not have to be necessarily taught by professors, you know? You can learn through your peers, and you [are] still gaining knowledge”* (Session 3, March 30, 2019).

### Call to Action #4: Accessible Communication and Creative Knowledge Translation

Our final call to action is about improving accessibility of DRR and climate change communication and knowledge translation, with a focus on equality and equity. Accessibility of preparedness resources and communication strategies, and inclusive services are all important to consider when enacting policy and practice changes. Two themes emerged during our discussions on communication in DRR and climate change: (1) The need for accessible community emergency resources; and (2) art as a tool for knowledge translation.

#### The Need for Accessible Community Emergency Resources

**Image 11.**
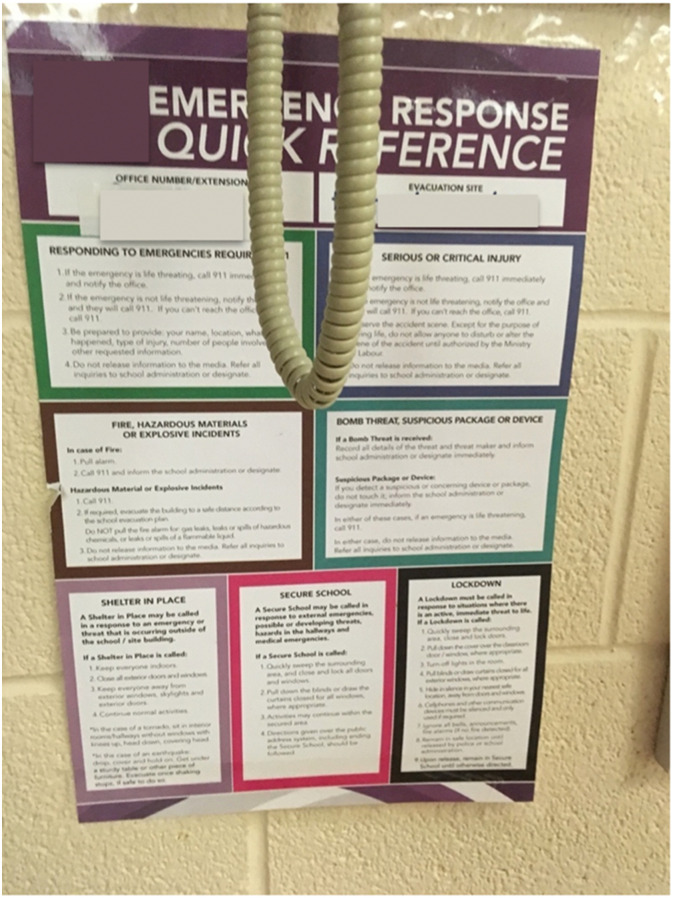
An emergency response poster in a high school classroom. The content is difficult to read due to the telephone cord hanging in front. The list of categories on the poster include the following: Shelter in place, responding to emergencies, serious critical injuries, fire, bomb threats, secure school, and lockdown.

Existing strategies to reduce disaster risks include using signs, posters, advertisements, and media campaigns to translate knowledge to citizens about what to do in disasters. While this is a good strategy, it should be done intentionally and communicated effectively to create behavior change. Content, audience, layout, and placement are all important factors to consider when creating accessible and useful informational posters, such as the one depicted in Image 11. Content needs to be easily understood and comprehensive without overwhelming the audience with information. Posters should be placed in a location with a clear view, unencumbered by objects blocking visual or physical access to the poster, such as telephone cords. The following quotations stem from our discussion about Image 11:

*“[The writing is] very tiny. Like you cannot take the time during an earthquake to [read what is on the poster]”* (Session 2, March 2, 2019).

*“They do not even go through these drills in the first place so how would you know what to do? Like would you just be standing there [reading the poster like,] ‘oh no an earthquake. No.’ and something topples on your head and you pass out?”* (Session 2, March 2, 2019).

Communication services such as news broadcasts, social media posts, pamphlets, and websites need to be universally accessible. Some examples of inclusive services are voice-to-text, T9-1-1, sign language interpreters, subtitles, and Braille. Unfortunately, these services are not always available. Before, during, or after a disaster, lack of such services, and poor quality of these services, can be fatal.

*“...it is so easy to get people to interpret properly like that is not a hard thing to do – there are so many interpreters. Then everything about disasters, a lot of it is just so hearing reliant.”* (Session 5, May 11, 2019).

*“It is not like people who are hard of hearing are rare either. There [are] a lot of Deaf people out there, there [are] a lot of blind people… even though people that can see and hear are the majority, you need to think of the minority, because maybe that deaf person knows something you do not, maybe that blind person can do something that you cannot …”* (Session 5, May 11, 2019).

**Image 12.**
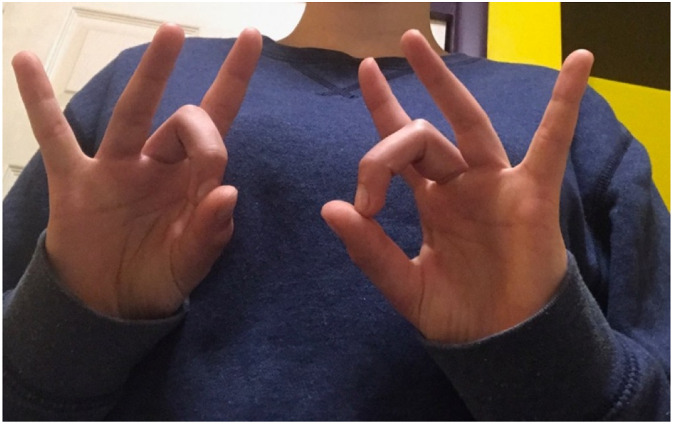
A youth co-researcher signing “disaster”.

Resources directing citizens about disaster risks and response procedures need to be accessible to the public. This means making citizens aware of the resources, while also educating them on how to use said resources. Access to community emergency and education resources contributes to improved preparedness, which leads to better adaptive capacity for the entire community (Image 12).

There are many facets that should be considered when discussing accessibility and DRR awareness. First, the accessibility of spaces and services for people in the community to access education around DRR. When discussing an art exhibit about earthquakes, one co-researcher had this to say about accessibility: “*...This gallery is in the middle of downtown and it is very accessible to see because the entire gallery is free…*” (Session 4, April 27, 2019). Here, we highlight geographic and monetary barriers in society to access DRR education. Money can also be a social barrier for citizens in creating grab bags, a suggested step from stakeholders for citizens to prepare for disasters: “*Everything disaster risk is expensive. Even something as simple as a grab bag takes money and effort to make*.” (Session 4, April 27, 2019).

#### Art as a Tool for Knowledge Translation

**Image 13.**
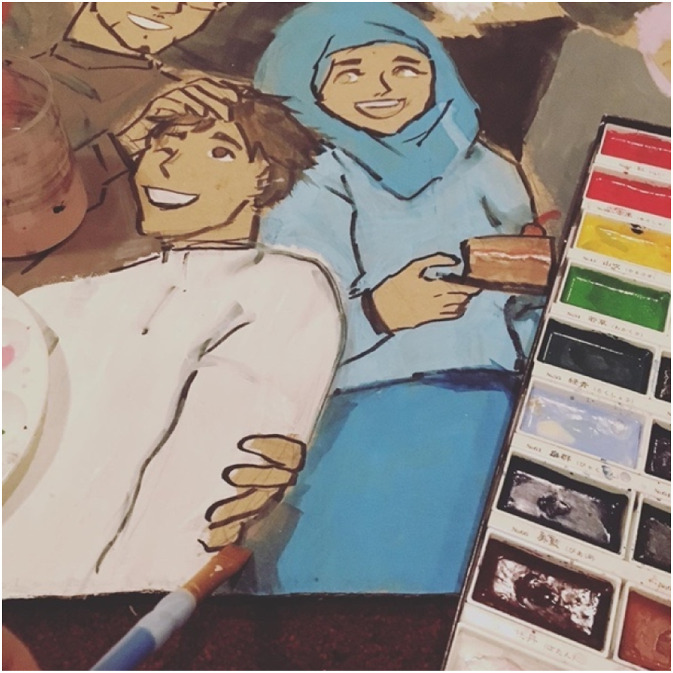
A youth co-researcher’s painting depicting diverse members of society in celebration of Eid. This picture represents the capability of art to cross cultural boundaries as an education and collaboration tool in DRR and climate change.

We believe that art is a powerful tool to connect with citizens and stakeholders; educate and create change in DRR and climate change; create cross cultural awareness; and promote inclusive collaboration. Image 13 shows an art piece which represents how art can cross multi-cultural boundaries to effect change.

Cultural constraints can make it difficult to have conversations on topics such as climate change. However, art is a tool that can cross cultural boundaries and effectively transmit “hard truths” about climate change. Every culture has different forms of artistic expression which can be harnessed to discuss difficult topics, facilitate inter- and intracultural communication, and relate to others on these issues.

*“There [are] a lot of cultures out there that kind of ignore the fact that our Earth is dying. If we talk about [it] with our parents about like ‘hey the earth is kind of dying’ they are like ‘oh no it is not. Stop being hooligans.’ Because they just choose to ignore it… The fact that you know the Earth is dying is not something that you want to think about. It is scary…”* (Session 6, June 1, 2019).

*“For hard-to-reach populations and for a lot of people, especially like Muslim culture, you will find a lot of paintings. In Iraq, you will find a lot of artists and people who generally enjoy art and putting message through art … you [could] probably do something [DRR or climate change related] into art and make it pleasing to look at. Maybe people will start doing something about [climate change].”* (Session 6, June 1, 2019).

**Image 14.**
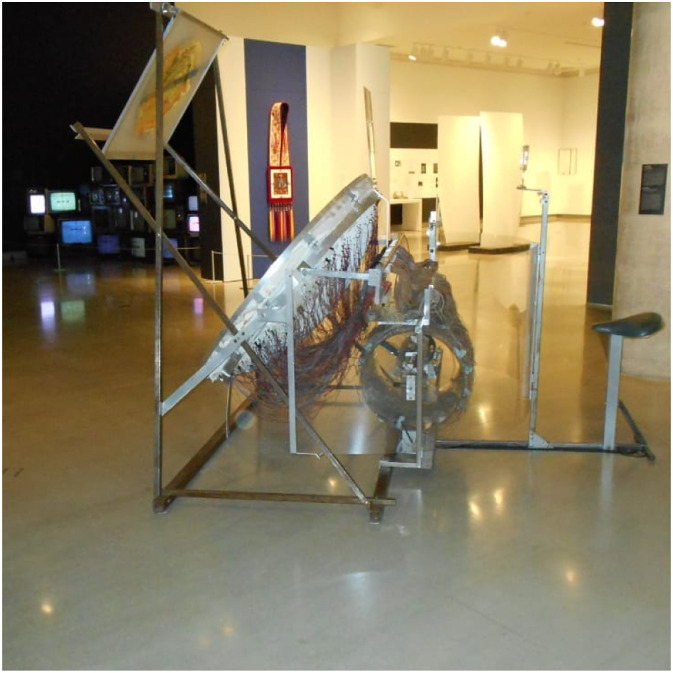
An art piece by Juan Geuer on display at the Ottawa Art Gallery representing how art and science can be combined to create exciting educational opportunities around DRR and climate change. (Art installation credit: Juan Geuer, Loom Drum, 1986–1992, Steel frame, Plexiglas, various materials, motor, lights, 12 volt circuits, map, lens system, Carbon + Light: Juan Geuer’s Luminous Precision at the Ottawa Art Gallery).

Art is an accessible medium to share information**.** DRR and climate change science can be combined with art, such is the case in Image 14, to spread awareness about disaster risk, disaster preparedness, and actions to combat climate change to a broad audience.

*“[This art piece] is from an art exhibit that I went to at the Ottawa Art Gallery. The artist[‘s] name is Juan Geuer and he uses both art and science to show emotion and spread information... He talks about sustainability in his artworks and [then there is this piece which is] really interactive about earthquakes as well. What I wanted to talk about was how art is a really good medium to share insight and information … it is really easy to show emotion and in turn because of this, people are more drawn to it.”* (Session 4, April 27, 2019).

Finally, we interpreted art as a collective and collaborative process enabling stakeholders to hear from the community and for citizens to communicate with each other, thereby promoting inclusive opportunities in DRR and climate change. Interactive art can enable social learning and create influence in DRR and climate change through opportunities for participation. This can help share knowledge and educate society using a fun, learn-by-doing, strategy.

## Discussion

The purpose of our Photovoice project was to explore youth perspectives on youth engagement in DRR and climate change, while connecting with stakeholders in the field. Our four calls to action can support DRR and climate change decision-making, policy, and practice.

Our first call to action is to include a diverse range of youth as key stakeholders in DRR and climate change strategies by harnessing their assets and building youth-adult partnerships. Youth are not a homogenous population; stakeholders should strive to create opportunities for a diverse group of youth to engage and collaborate on DRR and climate change decision-making, policy, and practice. These opportunities should be meaningful and avoid using the often applied top-down, patronizing, or tokenistic youth engagement strategies by adults, by asking youth how they want to participate (Bessant, 2004; Vromen, 2003). Additionally, these opportunities should use a youth development approach in which youth contribute to their communities and practice, while learning and growing in return (Cox et al., 2019; Pickering et al., 2021).

To support stakeholders in creating opportunities, and thus support the Sendai Framework, Paris Agreement, and SDGs in engaging in inclusive practices, stakeholders can also apply an asset-based approach. Morgan and Ziglio (2007) developed the asset model as a way to systematically apply an asset-based approach to population health issues and challenge the dominant deficit model. Asset models highlight capabilities, and needs, of people, environments, and contexts to tackle social inequities. Given that all three global agreements emphasize the importance of an equity lens, an asset-based approach would have stakeholders ask what capabilities youth have to support DRR and climate change —rather than focusing on their needs. In this way, and in consultation with youth, stakeholders can apply a bottom-up, youth-centered strategy to harness the power of youth to create change in these fields.

Youth engagement through volunteerism or work in DRR and climate change benefits society by contributing to social integration and cohesion (Reynolds, 2010), thus building social capital (Aldrich, 2015). The act of participation is also protective to the individual. There are many benefits to youth engagement in DRR, for instance, youth engagement in the creation and implementation of DRR initiatives can help youth learn about disaster preparedness while fostering teamwork, leadership, and social skills (Mahoney et al., 2003). In turn, youth represent a potential resource to help improve disaster preparedness in households and communities —and minimize the impacts when a disaster occurs.

Opportunities for youth participation should include learning opportunities, as the learning process builds confidence and skills, with long-term payout (Head, 2011). An example of a good practice supporting the SDGs and youth participation in DRR and climate change using learning processes is the Enhanced Rural Resilience in Yemen (ERRY) project (United Nations Department of Economic and Social Affairs, 2020). This project helped women and youth establish their own solar-powered micro-businesses by providing them with learning experiences on sustainable energy and entrepreneurship, as well as providing a stable income. In turn, the women and youth businesses supported the community and the environment by investing in local, self-reliant, sustainable energy sources to power their communities.

It is important to set deliberate and achievable goals to engage with hard-to-reach youth, for example, youth living in poverty, or refugees. Youth who already have knowledge, communication and organizational skills are more likely to engage in opportunities; this is an ongoing challenge for stakeholders in DRR and climate change, policy and practice (Head, 2011). As a population historically excluded from participation in policy and practice, youth are valuable assets to reach hard-to-reach youth and other populations. The Student Volunteer Army is an example of a DRR initiative engaging hard-to-reach youth. This initiative created opportunities for youth volunteers who are refugees to help with post-earthquake clean up after the earthquakes in Canterbury, New Zealand (Carlton, 2015).

A common thread across our calls to action is the importance of youth engagement in these improvements. Youth want opportunities for engagement; however, there is a gap in what we can do, what resources we have access to, and what we want to do—this is called a civic empowerment gap (Levinson, 2010). To reduce this gap, the power and resources of adults must be leveraged using youth-adult partnerships. Youth-adult partnership (Y-AP) is both a developmental process and a community practice, in which multiple generations collaborate to address common issues (Camino, 2000; Zeldin et al., 2012). Y-AP is a fundamental strategy to engage youth in DRR and climate change initiatives, mobilize assets, and promote positive youth development. Zeldin et al. (2012) identified authentic decision-making, mentorship, reciprocity, and community connectedness as central to Y-AP.

Youth are perceived as having weak political influence — not enough to make a difference in government decision-making (Frank, 2006). In the context of DRR and climate change, we want to see more Y-AP between politicians and youth. We would like to use the power that politicians have over policies, laws, and practice to create change that reflects diverse voices, including those of youth.

Y-AP between youth and politicians would be beneficial in reducing the ecological footprint of the fashion industry. Pre- and post-consumer, the fashion industry has a large ecological footprint (Dhir, 2021; Peters et al., 2021). Peters et al. (2021) found that most of the impacts on climate change stemming from the fashion industry occurred pre-consumer, in the clothing production and dissemination stages. We want to work with politicians to address SDG number 12 “responsible consumption and production” by reducing the fashion industry’s ecological footprint and switching to renewable energy (United Nations, 2015b).

Our second call to action is to use social media as a tool for social change with a direct impact in real life. Social media is a strong tool to complement or initiate community engagement in disaster preparedness initiatives, thereby supporting an all-of-society approach to DRR (UNDRR, 2015). It is an effective tool for DRR and climate change education, awareness and social change —but only if there is a direct impact offline. Youth are an important stakeholder with widespread expertise on social media, as well as trust in social media for news and informative learning compared to news media. Gatekeeping theory (Shoemaker, 2009) views people who decide what information gets messaged to society as gatekeepers. We view the select few with gatekeeping power in news media as biased and holding too much power for such a small amount of people. Social media helps to more evenly distribute the gatekeeping power, allowing our social networks to become additional gatekeepers (Bro & Wallberg, 2014).

Social media use needs to be informative, not performative, or risk losing societal trust. Performative activism, also known as performative allyship, occurs when members of nonmarginalized populations say they stand in solidarity with a marginalized population, but without follow through (Kalina, 2020). To dismantle oppressive societal power structures in DRR and climate change activism, antiracist and decolonial allies must avoid performative allyship. Performative allyship includes empty gestures (i.e., saying something with limited to no effect) (Blair, 2021). To support informative activism and solidarity, Blair (2021) suggests the following ethical approaches to solidarity and empty gestures: (1) Actions, defined by persons oppressed by societal power systems, are meant to dismantle oppressive power structures; (2) do research and learning prior to taking action; and (3) attention is on the act of disruption and not the person taking action. These meaningful actions can look like research, critical thinking, reflection, learning and unlearning, donating, and following Black, Indigenous, and People of Colour (BIPOC) and Indigenous leadership.

Our third call to action is to improve education curricula using multifaceted strategies to teach disaster preparedness and support community resilience at the individual, familial, and community levels. Current education on DRR is insufficient in Canada. In Ontario, we currently do not have a curriculum focused on DRR and community resilience. In the Ontario curriculum, grade nine geography has a dedicated disaster unit (Ontario Ministry of Education, 2018). Despite sections B1.1 to B1.5 dedicated to the physical environment and human activities, most of the focus is on risk, with minimal reference to the need for minimizing disaster impacts and disaster preparedness. We would like to see the following multifaceted curricular and extracurricular changes to improve disaster education in Ontario: (1) Curricular content on disaster preparedness and risk reduction; (2) more integration of DRR and climate change across different disciplines (e.g., math questions, English assignments, disaster management as a potential career path in Civics and Careers courses, etc.); (3) disaster and climate change course electives with student presentations and class projects on local disaster risks and preparedness, hands-on learning, and high quality teaching; and (4) extracurricular events, such as guest speakers, half-day school assemblies, disaster preparedness week, seminars and workshops, field trips, and clubs. This education is important to improve resilience to disasters, while reducing disaster risks.

Education and disaster awareness is yet another area in which youth can participate and lead. By engaging youth in knowledge creation and creating opportunities for participation in the field of DRR, we can become effective teachers and share our knowledge with those around us, including our parents (Ronan & Johnston, 2001). The literature shows that this knowledge exchange benefits us and supports DRR in our families and the wider community (Haynes & Tanner, 2015; Mitchell et al., 2008; Ronan et al., 2015; Tanner, 2010; UNICEF, 2012). This makes youth prime candidates for community leaders on disaster prevention/mitigation, preparedness, response and recovery.

Youth-led conferences are an example of an institutional space where youth can lead knowledge translation activities with confidence and gain leadership experience, and thus positive youth development (Larson, 2006; Lerner et al., 2011; Maton, 2008). For example, in 2015, 200 young professionals and students from across the world collaborated in the Children and Youth Forum at the Third UN World Conference on Disaster Risk Reduction (WCDRR) (Cumiskey et al., 2015). Youth were brought together by a youth-led organizing committee to exchange ideas on reducing disaster risks, building resilient communities, and advocating for the inclusion of youth priorities within the Sendai Framework. The participants said the conference was an excellent way to network with other young people and experts, build knowledge, and contribute to change (Cumiskey et al., 2015). Attendees emphasized the need for youth mentoring programs to continue post-conference, which emphasizes our own recommendation for Y-AP. These former participants, or any young person, could organize similar forums and conferences at local, provincial/territorial or national levels for youth.

Our fourth call to action is to improve accessibility of DRR and climate change communication and knowledge translation. Inclusive and universally accessible communication and language is a human right (United Nations, 2006), this includes disaster information sharing. Article 11 in the Convention on the Rights of Persons with Disabilities (CRPDs) stipulates that states take all necessary steps to ensure the safety of persons with disabilities in situations of risk, such as disasters (United Nations, 2006).

Inclusion is a guiding principle of DRR through promotion of empowerment and accessible and non-discriminatory all-of-society participation to achieve widespread engagement (UNDRR, 2015). In addition to accessible communication, stakeholders should also work to provide accessible DRR strategies. For example, not everyone has access to the resources (e.g., time, money, education) to create a 72-hr preparedness kit (Pickering et al., 2018). Populations also have the right to accessible emergency services through voice to text and T9-1-1. To create accessible DRR strategies, and inclusive communication campaigns, persons with disabilities, persons experiencing homelessness, persons living in poverty, youth, and other populations historically excluded from decision-making need opportunities to contribute to decision-making, policy, practice, and research.

The arts have the power to address issues upstream and make public health programs more accessible and equitable for diverse populations (Sonke et al., 2021). Not only should messaging be accessible, it should also be digestible and palatable for citizens. Compared to text, art can foster emotional responses to messaging (Lafrenière et al., 2014) and be a venue for meaningful and engaging dialogue which can influence behaviors, assumptions, and catalyze social change (Sonke et al., 2021). When combining arts and science as a strategy for knowledge translation it is important to collaborate with stakeholder groups to identify the need for an arts-based strategy, as well as what art form is most appropriate to convey the messaging (Archibald et al., 2018). Community art, art freely available in community spaces, can promote health, social cohesion and community resilience post disasters (Baumann et al., 2021). For instance, Baumann and Burke (2021) created a virtual art gallery for community members to submit artwork representing connection during the COVID-19 pandemic and found that the act of creating art itself had mental health and social benefits.

CBPR, such as Photovoice, is an effective tool to engage populations historically excluded from decision-making in deep, meaningful conversation, and contributions (Israel et al., 2012). As our Photovoice study demonstrated, we combined a variety of art mediums (i.e., photography, interactive art displays, lino block art, Word Art, and water color painting) to express ourselves on emotional and complex topics in DRR and climate change. Though we intended to only use photography, early in the project we began expressing ourselves using multiple art forms, triggering powerful engagement, and thus inspiring our theme “art as a tool for knowledge translation”. This freedom of self-expression enabled strong social connection internally (between our research team) and externally (with community partners and stakeholders), thereby promoting passionate dialogue and creative problem-solving, and sustainable opportunities for an all-of-society approach to DRR and climate change.

### Limitations

It is important to consider the findings of this study within the context of its limitations. While diverse in ethnicity, the small group all identified as female, were from the same city, and do well in academics. All participants had previous experience participating in a youth program focused on DRR; they were familiar with the researchers through this program. This recruitment limited the breadth of opinions captured in this study, but provided a strong foundation for rapport and engagement from the beginning of the project. Future studies are needed to include a diverse collection of youth voices on youth participation in DRR and reinforce further calls to action.

### Next Steps

Strong partnerships were formed between researchers, participants, and community partners during this study. We continue to collaborate through the EnRiCH Youth Research Team with the Canadian Red Cross to translate the knowledge gleaned from our study into action in DRR.

## Conclusion

Our study highlights the power of youth to design and implement inclusive, holistic, and multifaceted DRR and climate change strategies. Our Photovoice project provided an opportunity for high school and university students to collaborate and add our voices to DRR and climate change research. Youth are important stakeholders for inclusive, all-of-society participation—an integral pillar of the SDGs, Paris Agreement, and Sendai Framework—to reduce disaster risks and the effects of climate change. Building youth-adult partnerships and protecting inclusive policies and practice, youth, adults, and society can work together to change the current climate and disaster profile —and promote community resilience.
